# Screen-Printed Sensors Modified with Nafion and Mesoporous Carbon for Electrochemical Detection of Lead in Blood

**DOI:** 10.1149/1945-7111/ad2397

**Published:** 2024-02-13

**Authors:** Elena Boselli, Zhizhen Wu, Erin N. Haynes, Ian Papautsky

**Affiliations:** 1 Department of Biomedical Engineering, University of Illinois Chicago, Chicago, Illinois 60607, United States of America; 2 Departments of Epidemiology and Preventive Medicine and Environmental Health, University of Kentucky, Kentucky 40536, United States of America

**Keywords:** electrochemical sensor, lead detection, blood, screen-printed carbon

## Abstract

Lead (Pb) has long been acknowledged as a systemic toxicant, with pronounced health impacts observed even at low exposure levels, particularly in children. Adverse effects include diminished cognitive function, altered behavior, and developmental delays. Consequently, it is imperative to conduct regular monitoring of Blood Lead Levels (BLLs). In this work, we report on an electrochemical sensor based on screen-printed carbon electrode (SPCE) coated with Nafion and mesoporous carbon (MC). The sensor system uses simple sample preparation (acidification and dilution of whole blood), minimal sample volume (a few blood drops, 200 *μ*l), and swift time-to-results (1 h). A limit of quantitation (LOQ) of 0.3 *μ*g dL^−1^ Pb was achieved in whole blood. To demonstrate the practical utility of our sensor system, we evaluated its performance in the analysis of blood samples collected from children (n = 25). Comparative analysis with the laboratory-based gold standard method of inductively coupled plasma mass spectrometry (ICP-MS) demonstrated approximately 77% accuracy and 94% precision. We anticipate that our approach will serve as a valuable tool for more frequent BLL monitoring, particularly in communities where access to laboratory testing is impractical or expensive.

Lead (Pb) is a persistent heavy metal that does not naturally degrade in the environment and tends to accumulate in human tissues and organs.^1,2^ The primary sources of environmental Pb pollution have historically been anthropogenic emissions from industrial activities like mining and smelting.^1^ The exposure to Pb by general population primarily stems from Pb-containing products, such as leaded gasoline, plumbing pipes and fixtures, paint, and pesticides.^1,3^ Despite consistent efforts in the reduction of Pb exposure,^4,5^ significant elimination of Pb hazards remains a long-term challenge.^6,7^ Furthermore, Pb serves no physiological role in the human body and is recognized as a systemic toxicant.^8^ Pb exposure, even at low levels, is associated with adverse health effects spanning neurological, renal, hematological, cardiovascular, immunological, developmental, and reproductive impacts.^1,8,9^ Children are particularly vulnerable to Pb poisoning due to increased absorption rate and more frequent hand-to-mouth activity.^10,11^ Blood lead (BPb) levels have served as a prominent biomarker for Pb exposure.^1,9^ Importantly, there is no identified safe threshold for blood lead level (BLL), prompting regulatory agencies in the US and globally to continuously lower permissible levels of Pb in blood for children.^10^ These efforts have led to the current reference BLL of 3.5 *μ*g dL^−1^.^12^ Thus, widespread monitoring of BPb remains critically important, even at low levels, and especially among children.

Traditional analytical methods for detecting Pb in biological samples offer remarkable sensitivity, reaching detection limits as low as 0.01 *μ*g dL^−1^. However, they come with significant drawbacks. Methods like graphite furnace atomic absorption spectrometry (GFAAS),^13–15^ inductively coupled plasma atomic emission spectrometry (ICP-AES),^16^ and inductively coupled plasma mass spectrometry (ICP-MS)^17–19^ demand complex sample preparation procedures, are costly, and require highly-trained personnel to operate. These limitations confine these instruments to centralized labs and make them unsuitable for real-time analysis or frequent monitoring of Pb exposure. As an alternative, electrochemical techniques, such as anodic stripping voltammetry (ASV), have emerged as promising options for Pb detection due to their simplicity, cost-effectiveness, and portability. These techniques accumulate target analyte on the working electrode surface through electrodeposition, before sweeping potential more positively to strip the analyte off the electrode surface, generating a detectable faradaic current that correlates with the analyte concentration in the solution. Sensors based on materials like bismuth (Bi)^20–25^ or glassy carbon^26^ have been reported, with detection limits in the 0.1 *μ*g dL^−1^ (1 ppb) range. However, accurately quantifying Pb in blood samples without extensive sample preparation remains a challenge, primarily due to electrode surface fouling.

Enhancing the performance of electrochemical sensors, particularly in terms of selectivity and sensitivity, has been a subject of active research aimed at overcoming challenges associated with adsorption of organic components from the sample matrix onto the sensor surface. One effective strategy to enhance selectivity and mitigate fouling is to coat the working electrode (WE) with perm-selective membrane, such as Nafion.^27^ Nafion offers several advantages, including its perm-selectivity against organic surface-active compounds, chemical inertness, non-electroactivity, hydrophilicity, and ease of modification through the application of a thin layer via drop casting onto the electrode surface.^25–27^ For example, Hoyer et al*.* ^26^ demonstrated the benefits of coating thin-film Hg electrode with Nafion for detection of Pb using ASV in biological samples. On the other hand, nano/microstructuring of the WE increases the active surface area and enhances sensitivity. Various materials, such as carbon nanotubes, metal ion nanoparticles, and metal oxides have been explored for the effective detection of trace levels of Pb ions.^28–30^ However, these modifications often require expensive materials and intricate procedures. In contrast, mesoporous carbon (MC) has emerged as a noteworthy material for expanding the active surface area to enable more efficient electrochemical detection of metal ions.^31–35^ MC, characterized by pores within the 2–50 nm range, offers several advantages for electrochemical applications, including ease of electrode modification, cost-effectiveness, large specific surface area, strong adsorption capabilities, pore sizes optimal for improved mass transport of species, and electron transfer capabilities.^36–38^ Zhu et al.^35^ have leveraged MC/Nafion composites for sensitive electrochemical detection of Pb ions in tap water samples showcasing the successful amalgamation of mesoporous carbon properties and the cationic exchange capabilities of Nafion for trace-level heavy metal detection.

In this work, we report on an electrochemical sensor based on a screen-printed carbon electrode (SPCE) modified with Nafion and MC for detection of Pb in human blood without extensive sample preparation. Notably, this sensor offers high sensitivity, achieving a limit of detection (LOD) of 0.3 *μ*g dL^−1^ Pb in blood. To validate the sensor system, blood samples from a cohort (n = 25) of children living in the Southside of Chicago, IL, were analyzed. The sensor performance was benchmarked against the laboratory-based gold standard, ICP-MS, resulting in 77% accuracy and 94% precision. These findings indicate that our sensor system holds promise for point-of-use (POU) applications, enabling more frequent and widespread assessment of Pb exposure, particularly in vulnerable populations like children.

## Experimental

### Chemicals

Lead solutions of desired concentrations were made from AAS standard solution of 1000 mg l^−1^ Pb^2+^ in 2%–5% HNO_3_ (Sigma-Aldrich, St. Louis, MO). Hydrochloric acid (HCl) solutions were prepared by diluting 34 ∼ 37% HCl (Trace Metal Grade, Fisher Chemicals) with de-ionized (DI) water. Potassium chloride solution (1 M) for preparation of the Ag/AgCl reference electrode was made by dissolving crystalline KCl (Fisher Scientific) in DI water. Perfluorinated resin solution containing Nafion 1100 W (5% in low aliphatic alcohols) and mesoporous carbon nanopowder (<500 nm particle size) were purchased from Sigma Aldrich. Unless otherwise specified, all other chemicals were obtained from Fisher Scientific.

### Electrode modification

**Figure 1.**
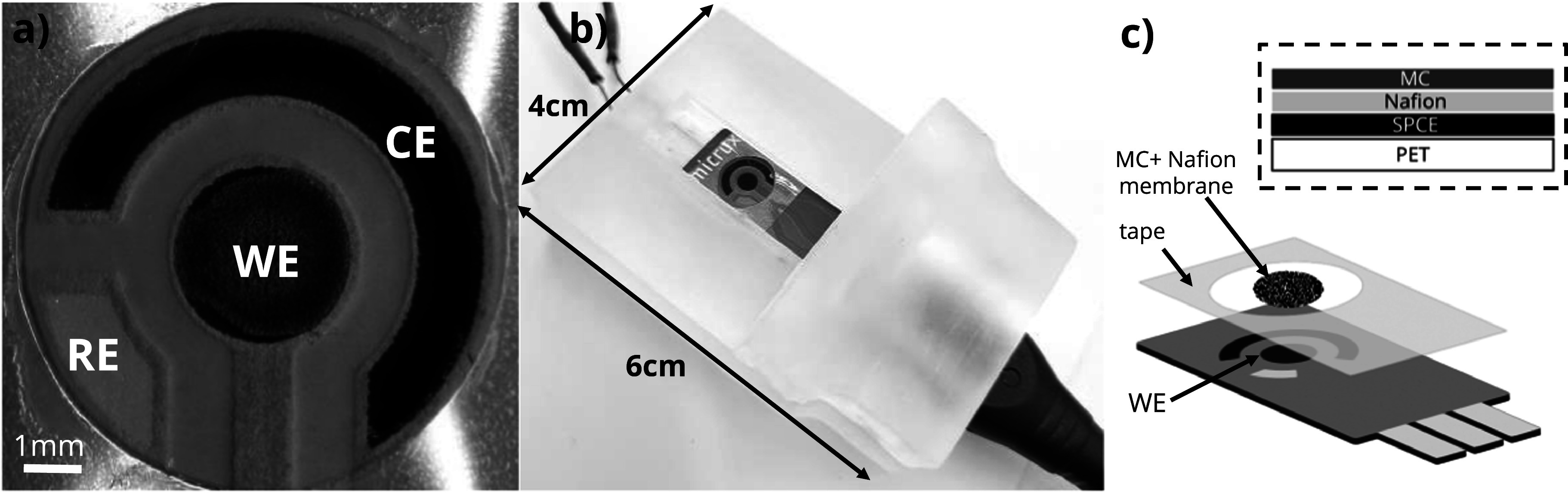
Electrochemical system for determination of Pb in blood. (a) Close-up view of the sensor with surface modified screen-printed carbon working electrode (WE), electroplated Ag/AgCl reference electrode (RE) and screen-printed counter electrode (CE). (b) Photograph of the sensor in 3D printed interface with miniature vibration motor and relative dimensions. (c) Schematic illustrating the structure of the sensor and composition of the MC + Nafion membrane in the highlighted area.

SPCE sensors (ED-S1PE-C, Micrux Technologies, Oviedo, Spain) on a PET substrate (350 *μ*m thick), feature a 3 mm carbon working electrode (WE), a silver reference electrode (RE), and a carbon counter electrode (CE). The RE was chloridized into silver/silver chloride (Ag/AgCl) with application of a constant current of 75 *μ*A (current density 2.5 mA cm^−2^) for 120 s in 1 M KCl. Figure 1a shows close-up of the sensor electrodes. The MC-Nafion composite solutions were obtained by dissolving 5 mg of MC nanopowder in 1 ml of 5% Nafion solution in low-aliphatic alcohols to achieve a concentration of 5 mg ml^−1^ MC. To promote even dispersion of the MC, solutions were ultrasonicated for 1 h prior to electrode coating. To coat the SPCE WE electrode, a 0.5 *μ*l droplet of the prepared MC-Nafion or Nafion solution was drop cast on the surface of the SPCE working electrode and allowed to dry for 1 h at a room temperature (∼20 °C) in ambient air. A layer of adhesive tape ∼60 *μ*m thick (3 M, Saint Paul, MN) was applied to achieve better confinement of the 45 *μ*L sample droplet onto the active electrode area. An 8 mm circular hole punch (Kucaa, Shenzhen, China) was used to punch the adhesive tape and define the sample area (Fig. 1b–1c). Electrochemical impedance spectroscopy (EIS) was used to characterize electrode coatings. For this, a potentiostat/ galvanostat (Reference 600+, Gamry, Warminster, PA) was used in the frequency range from 100 kHz to 0.01 Hz with 5 mM potassium ferricyanide (K_3_Fe (CN)_6_) as redox probe.

### Sensor system

The interface housing the sensor and the vibration motor was 3D printed in clear resin with a Form2 SLA printer (Formlabs, Somerville, MA). It consisted of the top piece (Fig. 1b), which housed a USB connector for the electrical connection, and the bottom piece, which housed a coin/disc microvibration motor (Digikey) for sample agitation. Postprocessing of the 3D printed components was done by ultrasonication in isopropanol (IPA) to remove all uncured resin and baked in the oven at 65 °C for 20 min to improve the mechanical strength. Wet sandpapers of 120, 320, and 800 grit were used progressively to remove support marks and to achieve smooth surfaces in printed structures. The interface was 6 cm × 4 cm. A USB-A cable was used to achieve electrical connection between the sensors and the potentiostat during measurement. The miniature vibration motor was 10 mm diameter and 2.7 mm thick and was operated via 3.0 V DC and 100 mA to vibrate at 12 000 rpm. It was powered and controlled via an Arduino Uno microcontroller (Arduino, Somerville, MA) to ensure uniform vibration only during the electrochemical deposition phase. We designed a simple PCB that assembled directly onto the ARDUINO board to drive the circuit. It comprised a switch to start the vibration and a potentiometer to precisely control voltage applied to the vibration motor.

### Sample preparation

Whole blood samples were collected from children aged 7 to 16 years in Southside Chicago, IL. The research study protocol and all study materials were approved by the Institutional Review Boards at University of Kentucky and University of Illinois Chicago. The blood samples were collected by venous blood draw in BD Vacutainer with K2 EDTA anticoagulant (lavender top-tubes), kept refrigerated at 4 °C, and delivered to the lab in batches weekly. Once received, samples were kept refrigerated at 4 °C until analysis. For electrochemical measurements, the samples were diluted 6x with 0.5 M HCl. Specifically, an aliquot of each received blood sample was transferred to a metal free conical tube (VWR, Radnor, PA) and 0.5 M HCl was then added to achieve a 1:6 ratio (v/v). Samples were stirred at 1000 rpm for 1 min, followed by electrochemical measurements. All samples were processed and analyzed within one week of the received date.

### Analytical procedure

Electrochemical measurements were performed using a portable potentiostat (EmStat3, Palmsens BV, Houthen, Netherlands). Cyclic voltammetry (CV) was performed to identify the potential window offered by the modified SPCE sensors and to locate the redox peaks associated with Pb in blood. Scan rate for CV was 100 mV s^−1^. Square wave ASV (SWASV) was used for detection of Pb. During deposition, Pb^2+^ was reduced to insoluble Pb^0^ and accumulated on the surface of the MC-Nafion modified working electrode at −1.1 V for 900 s, while the vibration motor provided stirring of the sample droplet. Stripping potential ranged from −1.1 V to −0.2 V, with square wave parameters of 50 mV amplitude, 3 mV step potential, and 30 ms period. For repeated measurements the electrode was cleaned by 10 cycles of CV in 0.1 M KCl. Linear calibration was obtained in the range from 0.3 *μ*g dL^−1^ to 10 *μ*g/dL. For LOD calculations, the 3*σ*/sensitivity method was used, with standard deviation (*σ*) at the lowest detectable Pb concentration (n = 30). A 3-points standard addition with +1 *μ*g dl^−1^, +3 *μ*g dL^−1^, +5 *μ*g/dL Pb spikes was performed for determination of the Pb concentration in the blood samples. Independent analysis of the blood samples was carried out using Thermo iCAP Q ICP-MS in the certified lab in New York state.

## Results and Discussion

### Optimization of sample conditions

**Figure 2.**
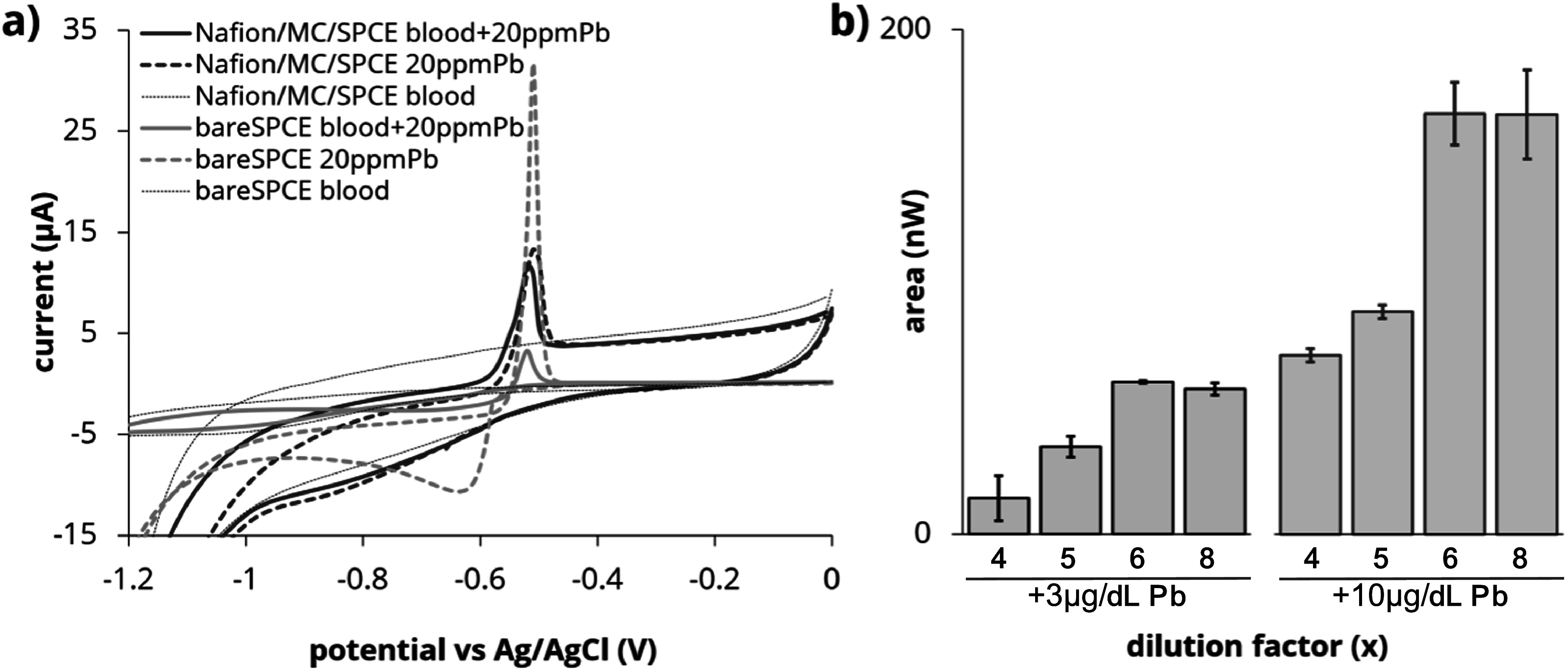
Initial performance of sensor in blood matrix. (a) Comparison of cyclic voltammetry on bare SPCE and Nafion/MC/SPCE in 0.5 M HCl electrolyte and blood 6x diluted with 0.5 M HCl*—*addition of 20 ppm Pb shows an anodic peak around −0.51 V. (b) Optimization of the dilution factor of blood in 0.5 M HCl on Nafion/MC/SPCE sensor at concentration spikes of +3 *μ*g dL^−1^ Pb (left, grey) and +10 *μ*g dL^−1^ Pb (right, blue). Deposition potential of −1.1 V and deposition time of 600 s were used.

Cyclic voltammetry (CV) was initially used to select the optimal supporting electrolyte for Pb detection on the SPCE sensor, assess the potential window of the sensor, and to identify the Pb redox peaks, in both the selected supporting electrolyte and representative blood sample. Hydrochloric acid (HCl) provided the highest anodic Pb peak in CV experiments when compared to sulfuric acid (H_2_SO_4_), nitric acid (HNO_3_), and acetate buffer (AB), as shown in Fig. S1. In addition, given its capacity to denature hemoglobin and consequently release Pb^2+^ ions from red blood cells (RBCs), HCl was selected as the supporting electrolyte for blood dilution and subsequent electrochemical measurements. Ionic strength and pH of HCl were assessed and optimal values of 0.5 M, pH = 0.5 were selected (Fig. S2).^15^ Figure 2a compares the voltammograms obtained for the bare SPCE and the SPCE coated with Nafion/MC in 0.5 M HCl, as well as in a representative blood sample diluted 6× with 0.5 M HCl (pH = 0.5). In the blood +0.5 M HCl background scan (dashed line), a large anodic current appears at potentials more negative than −1.2 V, signifying the onset of the hydrogen evolution reaction (HER) at the electrode at these acidic conditions. The addition of 20 ppm Pb results in a well-defined oxidation peak at approximately −0.51 V, consistent with prior literature.^31,39^ Notably, in this work, the modification of the WE surface does not impact the location of the Pb peak, which remains at −0.51 V in both 0.5 M HCl electrolyte and the diluted blood matrix. However, a 10-fold reduction in the magnitude of the Pb peak was observed for the bare SPCE (Fig. 2a, orange solid and dashed curves). Conversely, despite a 3-fold reduction in the magnitude of the Pb peak compared to the bare SPCE in 0.5 M HCl, no significant differences in performance between 0.5 M HCl and the blood matrix were observed on the Nafion/MC/SPCE (Fig. 2a, blue solid and dashed curves). These results highlight the ability of the Nafion/MC/SPCE to provide an appropriate potential window for Pb detection through SWASV and its superior performance in the more complex blood matrix compared to the bare SPCE.

A straightforward blood sample preparation method involving dilution with HCl was used in this work. Figure 2b presents optimization of the dilution factor within the range 4× to 8×. Dilutions below 4× produced inhomogeneous samples characterized by the presence of brown clusters upon visual inspection. This phenomenon might be attributed to an insufficient amount of HCl to lyse blood cells and release Pb^2+^ ions. Additionally, saturation of the SWASV signal was observed at dilutions greater than 6×. To confirm 6× as the most suitable sample dilution, evaluations were conducted at two different Pb concentrations, 3 *μ*g dL^−1^ and 10 *μ*g dL^−1^, which encompass the current blood lead reference value of 3.5 *μ*g dL^−1^.^12^ As Fig. 2b illustrates, a similar trend was observed at both 3 *μ*g dL^−1^ and 10 *μ*g dL^−1^ Pb concentrations. Thus, a 6× dilution in 0.5 M HCl was selected as the optimal sample preparation method for SWASV measurements.

Previous studies aiming to electrochemically detect heavy metals such as manganese (Mn), and zinc (Zn) in biological samples often necessitated acid digestion, wet ashing, or ultrafiltration to decomplex the target ions from organic compounds and proteins, making them available as free ions for electrochemical detection.^39–41^ However, in the case of Pb, which is not an essential trace element in the human body and is predominantly bound to hemoglobin in RBCs,^42^ dilution with 0.5 M HCl is the simplest procedure that allows for electrochemical detection of Pb^2+^ in blood.^15,43^

### Electrode surface modification

**Figure 3.**
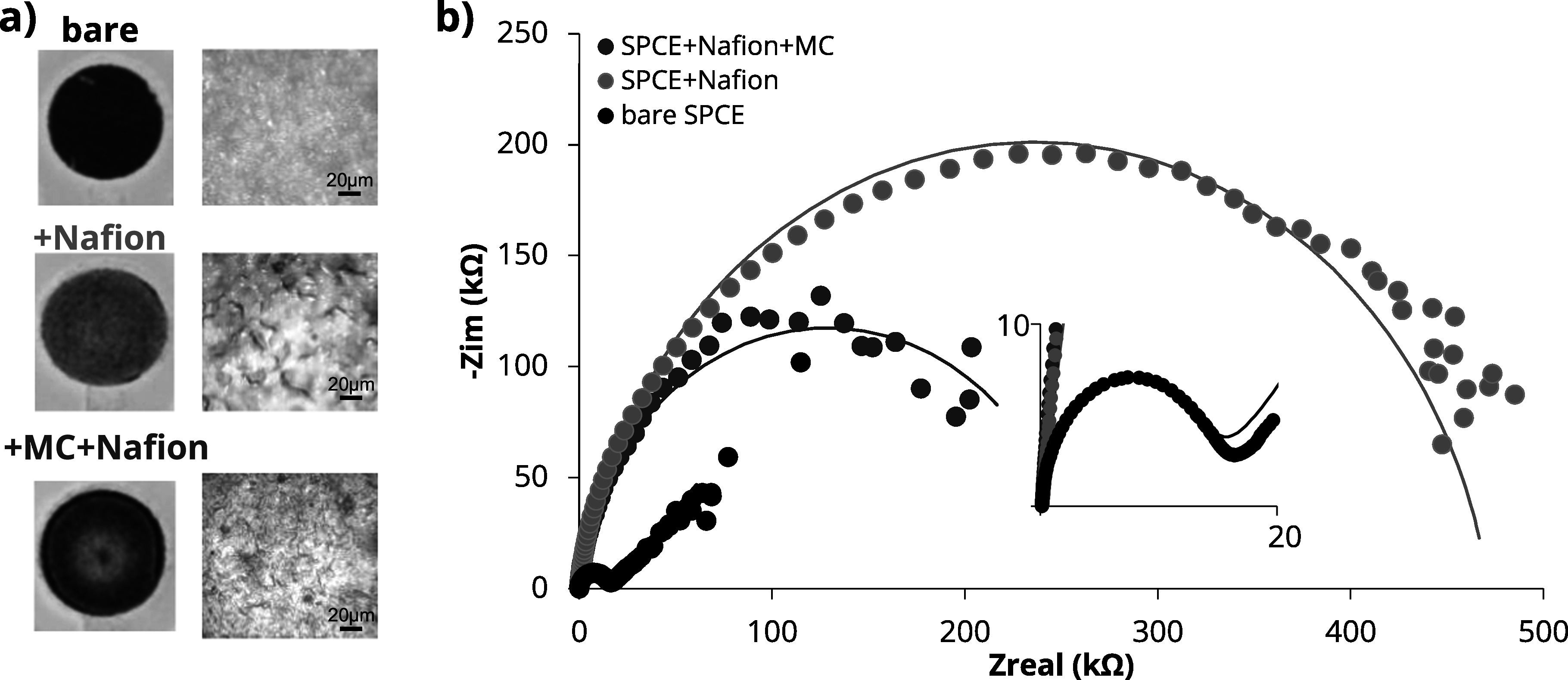
Characterization of SPCE WE surface modification. (a) Photographs of the modified WE illustrating addition of the Nafion and MC coatings. Zoomed panel indicates corresponding miscroscopic view of WE surfaces. (b) Corresponding EIS characterization of the WEs following surface modification. Inset illustrates close-up view of the bare WE in the lower impedance range.

In addition to identification of optimal sample conditions for Pb detection in blood on the modified SPCE, we also assessed and optimized electrode properties. The surface of the electrode under various conditions was first examined with an optical microscope to evaluate coverage of the WE with the Nafion and MC. As shown in Fig. 3a, coating the electrode with a 0.5 *μ*l droplet of Nafion film resulted in a uniformly duller appearance of the surface of the WE, indicating even coverage of the surface of the WE with the 0.5 *μ*l droplet of Nafion.^44^ However, after addition of MC, the electrode regained a vibrant black color with a well-defined lighter border, indicating the complete coverage of the WE by the Nafion/MC membrane (lower panel). A microscopic view, shown in the right panels in Fig. 3a, further confirmed coverage of the electrode with the Nafion membrane and the increased surface roughness following addition of MC. This feature provided greater active surface area and more coordinate sites for Pb deposition on the WE, and therefore was expected to improve sensitivity for Pb detection.^35^

To investigate the impact of surface modification on the electrical characteristics of the SPCE sensor, we used electrochemical impedance spectroscopy (EIS) to generate Nyquist plots for the unaltered (bare) SPCEs, as well as SPCEs coated with 5%Nafion and with or without 5 mg mL^−1^ MC. A 5 mM K_3_[Fe (CN)_6_] solution in 0.1 M KCl was used for this. The representative plots are shown in Fig. 3b, while the corresponding parameters of the EIS models and equivalent electrical circuits are reported in Table I. The unaltered (bare) SPCE exhibited an impedance equivalent to a constant phase element (CPE) with Warburg model, indicating diffusion-limited electron transfer at lower frequencies. The addition of 5% Nafion increased the charge-transfer resistance (R_ct_) and suppressed the diffusion limited kinetics at low frequencies (the CPE model fits the measured impedance, and there is no Warburg element). Indeed, the chemically resistant Nafion decreased the sensor’s efficiency in terms of the electron transfer kinetics, as evidenced by a ∼33.8× increase in the bare SPCE R_ct_ = 13.9 kΩ to the 5% Nafion modified SPCE R_ct_ = 471 kΩ. This result is consistent with the permselective properties of the Nafion, which hinder transport of negatively charged species, such as anions. Notably, the diameter of the semicircle in the Nyquist plot decreased with the introduction of MC on the modified electrode. This reduction in the electron transfer kinetics at the Nafion modified SPCE was behind our choice of further modification with MC. As reported in Table I, the addition of 5 mg ml^−1^ of MC partially recovered the electron transfer kinetics yielding R_ct_ = 257 kΩ for the 5 mg ml^−1^ MC + 5% Nafion modified SPCE. This is a ∼2× improvement in the charge transfer resistance compared to the SPCE modified with 5% Nafion only, suggesting an improvement in the electron transfer kinetics provided by the mesoporous carbon.

**Table I.** EIS parameters associated with different surface modifications of SPCE electrode.

WE	EIS fit model	Goodness of fit	Electrical parameters	Equivalent circuit
bare SPCE	CPE + diffusion (Warburg)	9.3 *10^−3^	R_s_ = 120.3 Ω	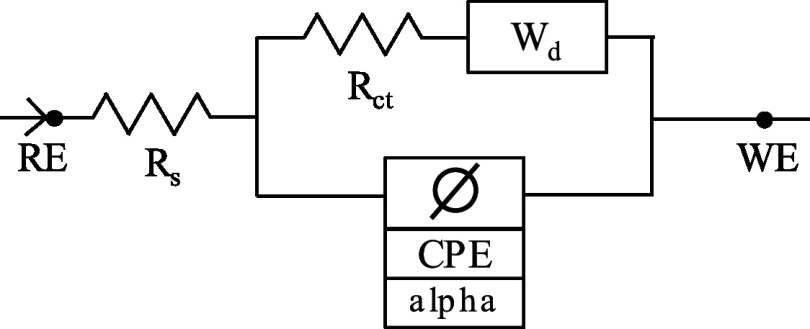
			R_ct_ = 13.9 kΩ	
			CPE = 0.26*10^−6 ^S*s^ɑ^	
			ɑ = 0.97	
			W_d_ = 60.2*10^−6 ^S*s^1/2^	
SPCE	CPE	3.9 *10^−3^	R_s_ = 127.2 Ω	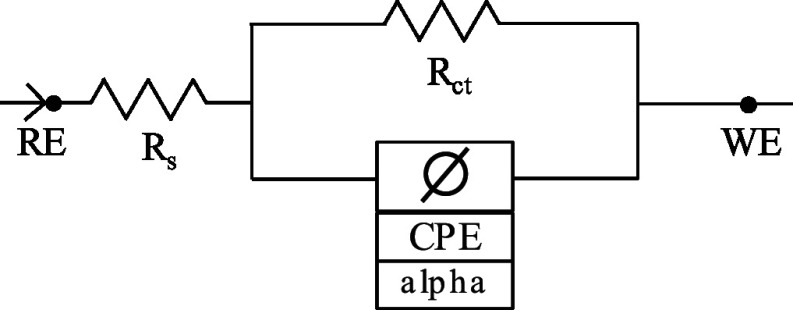
+ 5% Nafion			R_ct_ = 471.4 kΩ	
			CPE = 1.27*10^−6 ^S*s^ɑ^	
			ɑ = 0.89	
SPCE	CPE	1.4 *10^−3^	R_s_ = 122 Ω	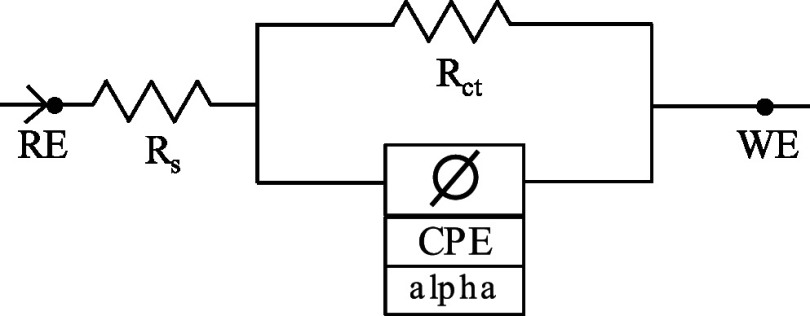
+ 5% Nafion			R_ct_ = 257 kΩ	
+ 5 mg mL^−1^ M^−1^C^−1^			CPE = 21.04*10^−6 ^S*s^ɑ^	
			ɑ = 0.94	

**Figure 4.**
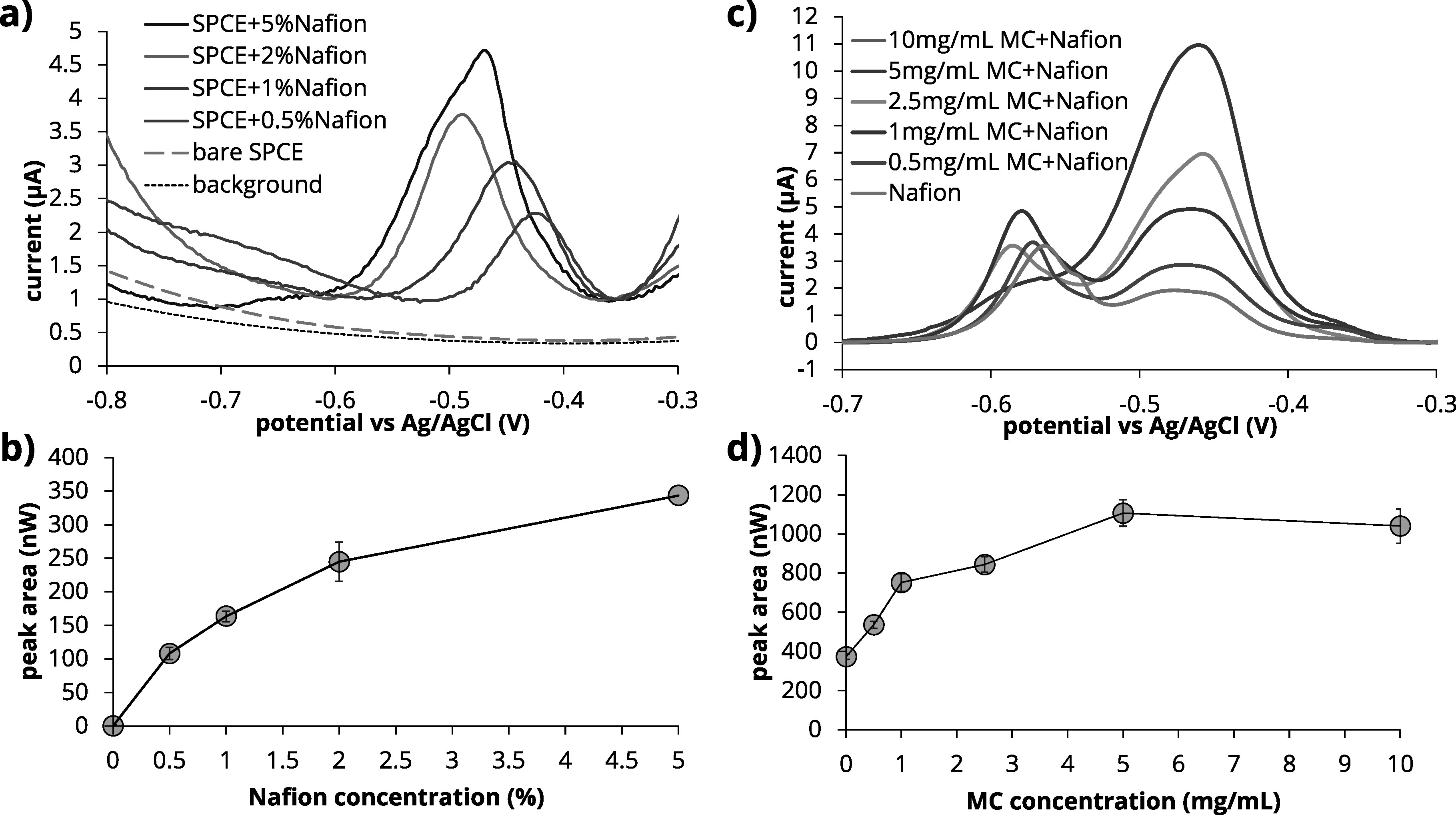
Optimization of surface modification of screen-printed carbon (SPCE) working electrode. (a) Optimization of Nafion concentration. Voltammograms in blood matrix on SPCE at different Nafion concentrations (b) Corresponding aggregate data (Pb concentration = 10 *μ*g dL^−1^). (c) Optimization of mesoporous carbon (MC) concentrations at 5% Nafion concentration. Voltammograms in blood matrix at different MC concentrations. (d) Corresponding aggregate data (Pb concentration = 10 *μ*g dL^−1^).

We optimized the WE surface modification using matched-matrix ASV experiments in spiked blood. First, we investigated the effect of Nafion concentration. As shown in the voltammograms in Fig. 4a and summarized in Fig. 4b, increasing the concentration of Nafion resulted in an increase in the signal associated with the 1 mg dL^−1^ Pb spike. These improved results can be attributed to a growing anti-biofouling capacity provided by the cation exchanging Nafion membrane, which facilitates the accumulation of metal cations, such as Pb^2+^, on the electrode surface at lower thicknesses and repels negatively charged species.^25,26^ On the other hand, excessive thickness of Nafion may impede the mass transport of the analyte^26^ and has been reported to cause cracks in the film due to contractile forces within the film itself.^45^ As Fig. 4b illustrates, an initial increase in Nafion film thickness yields an increased signal, but begins to saturate above 2%, indicating the onset of mass transport limitation of the Pb cations. The shift of the Pb peak potential to more negative values, from E_p_ ∼ −430 mV for 0.5% Nafion to E_p_ ∼ −500 m V for 2% Nafion can also be attributed to more facile stripping of Pb deposited on the electrode surface back into solution. At Nafion concentration of 5%, the potential of Pb peak shifts again to less negative potentials (E_p_ ∼ −490 mV), further supporting the notion of maximally enhanced mass transport. However, a 40% signal increase is still achieved from a 2% to a 5% Nafion concentration. Therefore, a Nafion concentration of 5% was selected for subsequent analysis.

To identify the optimal concentration of MC, we performed ASV in blood samples spiked with 10 *μ*g dL^−1^ Pb. Addition of MC provided enlarged active surface area and coordination sites available to favor Pb deposition on the WE.^35^ As Figs. 4c–4d illustrates, increasing the concentration of MC led to an increase in the stripping signal, reaching an optimal concentration at 5 mg mL^−1^ MC. Higher concentrations did not yield additional benefits. Visual inspection of the MC/Nafion solution suggested that concentrations >5 mg mL^−1^ resulted in incomplete dispersion of MC in the Nafion resin. Double peaks observed in the voltammograms are attributed to the nature of the metal deposits on the modified electrode surface, leading to differences in the thermodynamics of the stripping process.^46,47^ Due to the complex and broad shape of the voltammograms, area under the voltammogram was considered as relevant analytical signal for Pb^2+^ quantification. Both SWASV and EIS characterization demonstrate that SPCE modified with 5%Nafion+5 mg mL^−1^ MC offered the most favorable sensing surface for Pb detection in the blood matrix.

### Optimization of SWASV conditions for Pb detection in blood

In SWASV of this work, the redox reaction relevant for the electrochemical detection of Pb involves a 2-electron transfer during the deposition step, with the following reduction reaction at the surface of the electrode: ${{\mathrm{Pb}}}_{{\mathrm{aq}}}^{2+}$ + 2e^−^ → Pb^(0)^. Conversely, during the square wave stripping step, the following oxidation reaction occurs: Pb^(0)^ → ${{\mathrm{Pb}}}_{{\mathrm{aq}}}^{2+}$ + 2e^−^, where the previously deposited Pb^(0)^ is stripped back from the surface of the electrode into its ionic form. Heterogeneity of the SPCE surface has been suggested to offer sites of different strengths for Pb deposition on the electrode surface.^48^ Accumulation of Pb^2+^ on top of the previously deposited Pb^(0)^ gives rise to a multilayer structure that is hypothesized to be behind the complex peaks associated with anodic stripping of Pb in this work, as well as a number of previous studies.^49–51^

**Figure 5.**
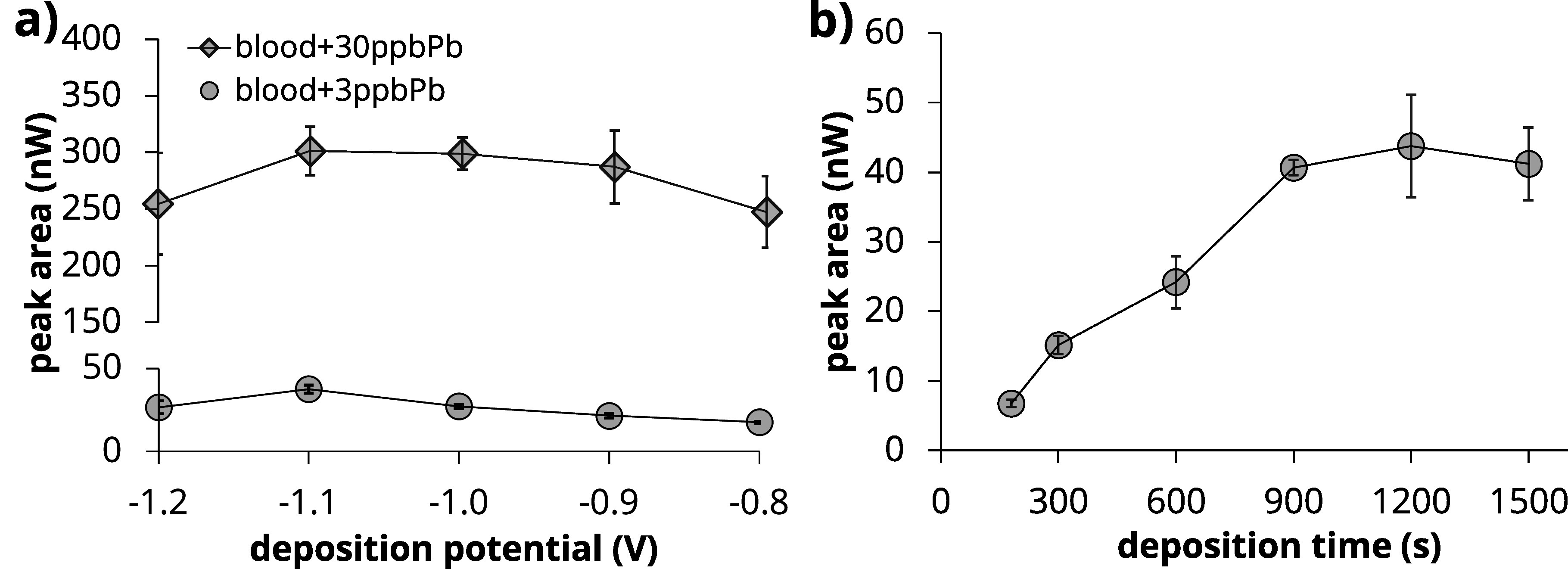
Optimization of parameters during the deposition phase of SWASV in blood matrix. (a) Optimization of deposition potential (−1.1 V selected). (b) Optimization of deposition time (900 s selected).

The selection of SWASV in this work was driven by several advantageous characteristics. First, the “*built-in”* preconcentration capability of stripping voltammetry allows accumulation of Pb onto the electrode surface and its detection at trace levels.^52–54^ Second, the square wave voltammetric waveform applied to the WE during the stripping step allows for better background current rejection and reduced interference of dissolved oxygen.^52,55,56^ We then evaluated the effects of deposition potential and time in spiked blood samples as most relevant factors for SWASV optimal performance. As shown in Fig. 5a, a more negative potential resulted in improved deposition efficiency of Pb. Two different concentrations were used in the optimization—an easily detectable spike of +10 *μ*g dL^−1^ Pb (+100 ppb), and a spike of +3 *μ*g dL^−1^ Pb (+30 ppb), since the current BLRV is 3.5 *μ*g dL^−1^ Pb. Specifically, there was an increase in the Pb stripping peak when the deposition potential varies between −1.2 V to −0.8 V, which is consistent with the previous literature on Pb detection with Nafion-modified carbon electrodes in a variety of sample matrices.^35,40^ However, at potentials more negative than −1.2 V (vs Ag/AgCl RE), the hydrogen evolution became significant^57^ and introduced variability in the Pb stripping signal without improving the signal-to-noise ratio. A deposition potential of −1.1 V provided the optimal signal-to-noise ratio, especially at low +0.3 *μ*g dL^−1^ Pb spikes, with a ∼38% increase when comparing deposition potential of −1.1 V vs −1 V.

The effect of deposition time is reported in Fig. 5b. Longer deposition times permitted more Pb to accumulate on the WE surface. However, excessively long deposition times led to sample drying, resulting in no improvement in sensitivity. This was evident from increased variability and signal saturation at deposition times exceeding 900 s. Thus, a value of 900 s was used in this work.

### Analytical performance of the modified sensor

**Figure 6.**
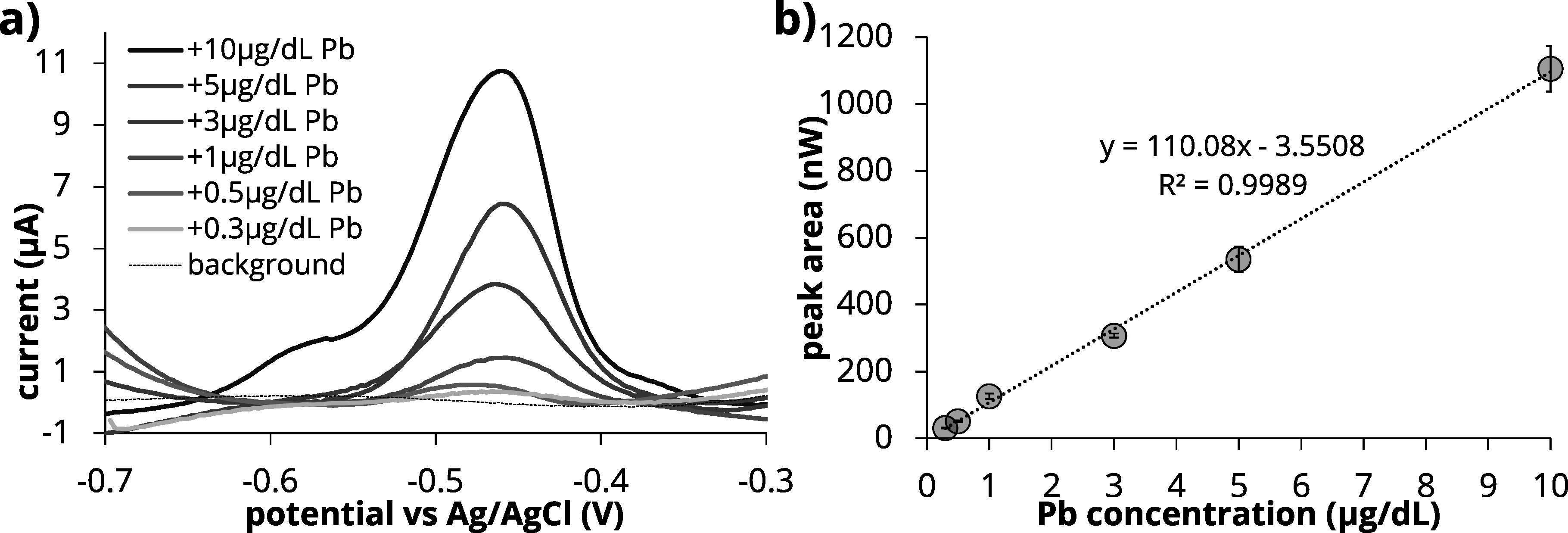
Performance of the system in blood matrix diluted 6x with 0.5 M HCl. (a) calibration curve Pb concentration 3 ppb-100 ppb comparison of Nafion only vs Nafion+ MC over the physiologically relevant Pb concentration range. (b) Corresponding voltammograms obtained on SPCE/Nafion/MC modified electrode. SWASV parameters as follows: deposition potential = −1.1 V, deposition time = 900 s, step increment = 3 mV, square wave period = 30 ms, square wave amplitude = 50 mV.

After optimizing the experimental conditions, we assessed the analytical performance of the sensor system. A calibration curve was constructed within the range of 0.3 to 10 *μ*g dL^−1^ Pb in blood diluted 6× with 0.5 M HCl. This range encompasses the current blood reference level of 3.5 *μ*g dL^−1^ Pb (35 ppb Pb) in children.^12^ As shown in Fig. 6a, at Pb concentrations >5 *μ*g dL^−1^ a shoulder appears on the Pb peak at a stripping potential of −0.6 V. This has been reported in previous work by our group for ASV of Pb on copper (Cu) WE and was attributed to the deposition of a thin layer of Pb over Pb itself rather than on the electrode surface, leading to more facile stripping starting at more negative potentials.^58^ This complexity of the voltammograms supports the use of peak area in place of peak magnitude for Pb quantification.^59^

The correlation equation for peak area measurements shows a sensitivity of 110 [nW/(*μ*g/dL)] and linearity R^2^ = 0.998 in the range 0.3–10 *μ*g dL^−1^ Pb. Notably, the calculated limit of detection (LOD) considering the required 6x dilution is 0.39 *μ*g dL^−1^ based on the 3*σ*/slope (n = 10), with *σ* equal to the standard deviation at the limit of quantitation (LOQ) of 0.3 *μ*g dL^−1^ Pb. Importantly, the reported limit of detection is below the current safety BLL of 3.5 *μ*g dL^−1^.

This performance is compared with prior studies on electrochemical detection of Pb in human blood matrix in Table II. Despite a higher LOD compared to the one on Hg-based electrodes,^60,61^ toxicity of Hg makes these sensors impractical for field applications. Nafion/MC/SPCE therefore provides a safer alternative. Conversely, Nafion/MC/SPCE achieves linear range in the same order of magnitude and extent as SPE modified with more sophisticated sensor fabrication protocols^62^ or use of more expensive materials such as gold-based electrodes.^63^ In addition, the achieved LOD is a ∼2× improvement compared to previous studies on boron doped diamond (BDD) coupled with complex sample preparation procedures.^44,64,65^ Additional examples in recent literature on Pb stripping have focused on environmental samples.^66,67^

**Table II.** Previous works on electrochemical detection of Pb in human blood matrix.

References	Sensor	Technique *	Sample preparation	Gold standard **	Calibration range (*μ*g/dL)	LOD (*μ*g/dL)
Li et al.^54^	cyclodextrin modified gold electrode (MEA-*β*-CD)	DPSV	heating at 100 °C with HNO_3_	ICP-AES	0.35 −19.2	0.14 (standard solutions 1 M HClO_4_)
			(3.5x dilution)			
			addition of DI H_2_O			
			10x dilution with			
			1 M HClO_4_			
Zhao et al*.* ^53^	mesoporous polymer of melamine-formaldehyde/SPE	SWASV	not reported	AAS (*n* = 6 blood samples)	0.1 − 5	0.1
Yantassee et al*.* ^52^	Hg-film on glassy carbon WE	FIA / ASV	ultrafiltration	ICP-MS	0 − 2.0	0.046%RSD = 2.4 (10%blood)
			(protein removal)			
			dilution to 20%blood with 1 M HCl			
			(5x dilution)			
			2x dilution to 10%blood			
			(0.5 M HCl)			
Mai et al*.* ^58^	glassy carbon WE	DPV / Cu^2+^ enhancement (800 ppb Cu^2+^)	3x dilution HNO_3_	AAS (*n* = 5 blood samples)	0.2 −10.0 (standard 0.01 M HNO_3_)	0.1%RSD = 4.6 (standard 0.01 M HNO_3_)
			heating at 100 °C			
			additional dilution HNO_3_			
Kruusma et al*.* ^55^	bismuth-film modified boron-doped diamond WE (Bi-BDD)	sono-electroanalytical SWASV + ultrasound-assisted deposition	pretreatment: 2/3 dilution,centrifugation	AAS	0.62 − 8.9	0.87 (2%blood, 0.1 M HNO_3_ pH0.9)
			50x dilution in 0.1 M HNO_3_ pH0.9, (2%blood)			
Benzhi et al*.* ^34^	Nafion coated Bismuth-film WE	DPSV	pretreatment with HNO_3_ acidification	AAS (*n* = 2 blood samples)	0.17 − 2.79 (standard 0.1 M AB)	0.013%RSD = 3.1 (standard 0.1 M AB)
			+ advanced oxidation process (AOP with H2O2 and UV irradiation)			
Bannon et al*.* ^15^	Hg-coated graphite WE (ESA Trace Metal Analyzer)	ASV	dilution with reagent to 3.33% blood	GFAAS	1.0 − 70.0 (3.33% blood)	1.0%RSD = 11 (3.33% blood in reagent, pH = 1.3–1.4)
			(30x dilution, 100 *μ*L blood in 3000 *μ*L solution)			
Feeney et al*.* ^43^	LeadCare II	ASV	dilution with proprietory treatment reagent (containing HCl)	GFAAS	3.3 − 65	3.3
this work	Nafion/MC/SPCE WE	SWASV	6x dilution with 0.5 M HCl	ICP-MS	0.3 −10	0.39

**Figure 7.**
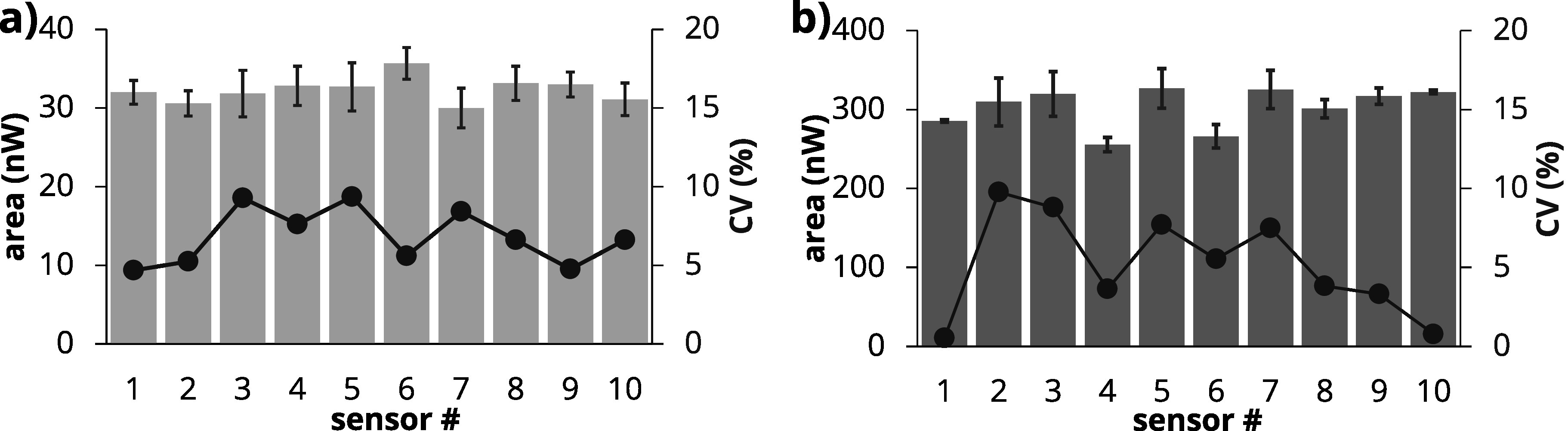
Performance of the sensor system in blood matrix diluted 6x with 0.5 M HCl. (a) Reproducibility at 0.3 *μ*g dL^−1^ Pb in blood matrix (n = 3 repetitions for each sensor). (b) Reproducibility at 3 *μ*g dL^−1^ Pb (n = 3 repetitions for each sensor). In both panels, stripping peak area is plotted on the primary (left) axis, while the variability of each sensor is plotted on the secondary (right) axis.

To evaluate the reproducibility and precision of the measurements, repeated SWASV was performed at 0.3 *μ*g dL^−1^ and at 3 *μ*g dL^−1^ of Pb using n = 10 different sensors, with 3 repetitions for each sensor. The overall reproducibility at both of the tested concentrations yielded an average coefficient of variation (CV) of 7.5 ± 1.8%. Figure 7a illustrates the stripping peak areas, plotted on the primary (left) axis, and the variability of each sensor, plotted on the secondary (right) axis, at 0.3 *μ*g dL^−1^ Pb in blood. Similar experiments were performed at a higher concentration of 3 *μ*g dL^−1^ Pb (Fig. 7b). Results showed an increase in variability at the higher Pb concentration, with an average CV of 9.6 $\pm $ 3.2%. Thus, the sensor precision was calculated as ∼90%. These results are comparable to our previous work on detection of Mn in water.^47^

Interference from metal ions present in human blood, such as Mn and Zn, can potentially impact sensor performance. However, in a blood matrix most other metals, including Mn and Zn are tightly bound to proteins and are reported to require more complex extraction procedures such as acid or microwave digestion prior to their electrochemical determination.^41,68,69^ Moreover, the existing literature reports no interference due to Mn and Zn in voltammetric detection of Pb on screen-printed carbon electrodes and MC / Nafion composite on glassy carbon electrodes.^35,48^ In turn, stripping of Mn and Zn occurs at more negative potentials, outside the potential window provided by the carbon based electrodes.^70^ Thus, these metals are not expected to interfere with the determination of Pb in blood.

### Determination of Pb in blood

**Figure 8.**
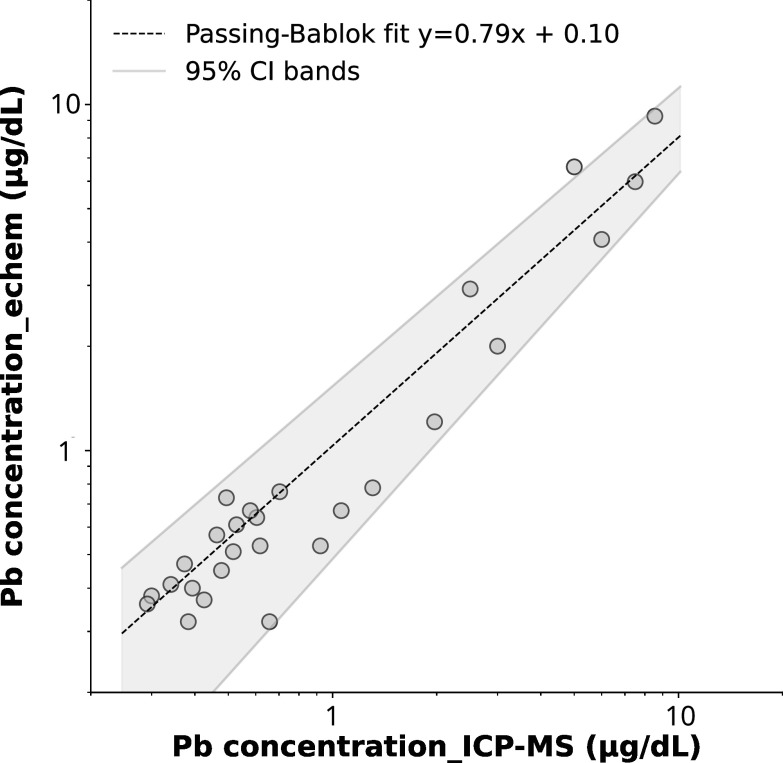
Passing-Bablok analysis for comparison of ICP-MS vs electrochemical method (this work) for n = 30 subject blood samples. Dashed line represents regression line. Shaded area identifies the 95% confidence interval (CI) bands; upper 95% CI: 1.09 + 0.19, lower 95%CI: 0.64x—0.05.

We validated sensor performance with venous blood samples from a cohort of children. A total of n = 30 samples was tested, and electrochemical results were benchmarked against ICP-MS from a certified laboratory for trace metal analysis. Standard addition procedure with 3 added spikes is implemented to overcome potential effects arising from the different compositions of complex biological matrices. Only one sample presented Pb concentration above the current reference value of 3.5 *μ*g dL^−1^.^12^ To test the performance of our approach in a broader range of Pb concentrations, blind spiked experiments were performed (n = 5). The Pb concentration range tested is 0.3–10 *μ*g dL^−1^ Pb. As reported in Table III, an average accuracy of ∼77% is obtained when benchmarked against ICP-MS results from a certified laboratory analysis. Considering the LOQ of 0.3 *μ*g dL^−1^ as threshold, the calculated concordance/agreement defined according to conventional statistics as TP+TN/n is 87.1%. Passing-Bablok regression was implemented to evaluate agreement between the electrochemical results and ICP-MS results, and to identify presence of proportional or constant bias between the two analytical methods.^71,72^ Results for the slope and intercept for the Passing-Bablok regression are reported in Fig. 8 and summarized in Table III with their 95% confidence intervals (95% CI). Since 95% CI of slope contains value 1 (1.09–0.64), it can be concluded that there is no proportional bias between the two methods. On the other side, 95% CI of intercept contains value 0 (0.19 – −0.05) and therefore no constant bias between the two methods is present. This analysis further corroborates the usefulness of Nafion/MC/SPCE for electrochemical detection of Pb in blood suitable for point-of-care applications.

**Table III.** Comparison of electrochemical analysis vs ICP-MS analysis from certified laboratory for the measurement of Pb in blood samples (n = 30).

Statistical index	Value
Mean accuracy detected samples (%)	(1 − E__rel__) * 100 = 77.1%
Mean precision detected samples (%)	(1 − CV) * 100 = 94.0%
Concordance/agreement (%)	$\frac{{\mathrm{TP}}+{\mathrm{TN}}}{{\mathrm{n}}}\,* $ 100 = 87.1%
Passing-Bablok regression slope	0.79 (95%*CI* 1.09–0.64)
Passing-Bablok regression intercept	0.10 (95%*CI* 0.19 – −0.05)
Pearson correlation coefficient	r = 0.959

## Conclusions

This work presents the characterization of a Nafion / MC SPCE for detection of Pb in blood. Tailored surface modification with Nafion and Mesoporous Carbon of SPCE allows to reach a detection limit of 0.39 *μ*g dL^−1^ Pb in blood matrix. Moreover, applicability of the system for detection of blood samples from children is demonstrated with 94% precision and ∼77% accuracy, compared with the ICP-MS gold standard measurements. Advantages of the proposed electrochemical system include simple sample preparation (6× dilution of whole blood), small total sample volume of few blood drops (200 *μ*l), short time to results (1 h), and compact format. Practical benefits include potential for more convenient, widespread and frequent monitoring of BLLs at the POC, especially in vulnerable populations such as children.
